# Strain diversity of *Treponema pallidum* subsp. *pertenue* suggests rare interspecies transmission in African nonhuman primates

**DOI:** 10.1038/s41598-019-50779-9

**Published:** 2019-10-02

**Authors:** Idrissa S. Chuma, Christian Roos, Anagaw Atickem, Torsten Bohm, D. Anthony Collins, Linda Grillová, Luisa K. Hallmaier-Wacker, Rudovick R. Kazwala, Julius D. Keyyu, Simone Lüert, Ulrich Maloueki, Jan Oppelt, Klára J. Petrželková, Alexander Piel, Fiona A. Stewart, David Šmajs, Sascha Knauf

**Affiliations:** 1 Work Group Neglected Tropical Diseases, Infection Biology Unit, German Primate Center, Leibniz Institute for Primate Research, Goettingen, Germany; 2 Primate Genetics Laboratory, German Primate Center, Leibniz Institute for Primate Research, Goettingen, Germany; 3 Department of Veterinary Medicine and Public Health, Sokoine University of Agriculture, Morogoro, Tanzania; 4 Gene Bank of Primates, German Primate Center, Leibniz Institute for Primate Research, Goettingen, Germany; 5 Department of Zoological Sciences, Addis Ababa University, Addis Ababa, Ethiopia; 6 African Parks, Odzala-Kokoua National Park, Brazzaville, Republic of the Congo; 7 Jane Goodall Institute, Kigoma, Tanzania; 8 Biology of Spirochetes Unit, Department of Microbiology, Institut Pasteur, Paris, France; 9 Department of Biology, Faculty of Medicine, Masaryk University, Brno, Czech Republic; 10 Tanzania Wildlife Research Institute, Arusha, Tanzania; 11 Department of Biology, Faculty of Sciences, Kinshasa University, Kinshasa, Democratic Republic of the Congo; 12 CEITEC-Central European Institute of Technology, Masaryk University, Brno, Czech Republic; 13 Institute of Vertebrate Biology, Czech Academy of Sciences, Brno, Czech Republic; 14 Institute of Parasitology, Biology Centre, Czech Academy of Sciences, Ceske Budejovice, Czech Republic; 15 Zoo Liberec, Liberec, Czech Republic; 16 School of Natural Sciences and Psychology, Liverpool John Moores University, Liverpool, UK; 17 Greater Mahale Ecosystem Research and Conservation Project, Dar es Salaam, Tanzania; 18 Division of Microbiology and Animal Hygiene, University of Goettingen, Goettingen, Germany

**Keywords:** Microbial ecology, Bacterial infection

## Abstract

In our most recent study, we found that in Tanzania infection with *Treponema pallidum* (*TP*) subsp. *pertenue* (*TPE*) is present in four different monkey species. In order to gain information on the diversity and epidemiological spread of the infection in Tanzanian nonhuman primates (NHP), we identified two suitable candidate genes for multi-locus sequence typing (MLST). We demonstrate the functionality of the MLST system in invasively and non-invasively collected samples. While we were not able to demonstrate frequent interspecies transmission of *TPE* in Tanzanian monkeys, our results show a clustering of *TPE* strains according to geography and not host species, which is suggestive for rare transmission events between different NHP species. In addition to the geographic stability, we describe the relative temporal stability of the strains infecting NHPs and identified multi-strain infection. Differences between *TPE* strains of NHP and human origin are highlighted. Our results show that antibiotic resistance does not occur in Tanzanian *TPE* strains of NHP origin.

## Introduction

Nonhuman primates (NHPs) in Africa are naturally infected with *Treponema pallidum* subsp. *pertenue* (*TPE*)^1^, the bacterium causing human yaws. In our most recent study, we found that in Tanzania infection with *TPE* is present in four different NHP species (olive baboon (*Papio anubis*), yellow baboon (*Papio cynocephalus*), vervet monkey (*Chlorocebus pygerythrus*), and blue monkey (*Cercopithecus mitis*))^2^. Moreover, we showed that infection is geographically widespread within Tanzania. Although we confirmed infection by serology and PCR, the data were insufficient to describe the epidemiology of the disease. Further insights into the inter- and intraspecies spread of the *TPE* bacterium in Tanzanian NHPs will contribute to our understanding of transmission pathways and pathogen maintenance, which are crucial elements for the identification of a functional disease reservoir^3^. The chance that NHPs infected with *TPE* are a potential source for human infection has been discussed for tropical Africa^4^. However, naturally occurring transmission from NHPs to humans and *vice versa* has not been confirmed by current data, although phylogenetic analyses of whole genome sequences from *TPE*s of human and NHP origin suggest a rapid initial radiation of the ancestor of *TPE* across the different primate taxa, including humans^1^.

Molecular typing is used to accurately distinguish between different strains of *T*. *pallidum* (*TP*) for epidemiological and surveillance analysis. The method has been extensively applied to the syphilis–causing bacterium (subsp. *pallidum*, *TPA*)^5^ where it is used to describe its spatial, e.g.^6–9^, and temporal, e.g.^10^, subtype composition. Two recent studies suggested new typing systems for human yaws^11,12^. It was unclear though, whether the existing molecular typing systems for *TPA* or *TPE* of human origin can be applied to *TP* strains originating from NHPs.

In the current study, we identified suitable candidate genes for multi-locus sequence typing (MLST) in *TP* samples of NHP origin and investigated strain diversity of the NHP infecting strains in Tanzania. We hypothesized that interspecies transmission in NHPs is ongoing. Moreover, we show that our typing system can be applied to samples from other regions of Africa and to analyze *TP* in non-invasively collected fecal samples.

## Materials and Methods

### Ethical statement

No animals were handled specifically for this study. The ethical statement for the Tanzanian NHP samples has been published elsewhere^2,13^. Lesion swabs from Ethiopian grivet monkeys (*Chlorocebus aethiops*) were taken as part of a research investigation conducted by AA under the Ethiopian Wildlife Conservation Authority reference number 15ET-0000-BS-01. Noninvasively collected fecal samples of western lowland gorillas (*Gorilla gorilla gorilla*) from the southern part of Odzala-Kokoua National Park (OKNP) originate from a collaboration signed under a MoU between the Foundation Odzala-Kokoua, the German Primate Center, and the Institute of Vertebrate Biology, Czech Academy of Sciences in November 2017.

### Design of the multi-locus sequence typing system

In order to identify most suitable candidate genes for MLST in *TPE* strains of NHP origin, we used 23 available complete and draft genome sequences of *TPE* from both human and NHPs from Africa and the Pacific regions (Table S1). Several criteria were applied to obtain most suitable gene loci for *TPE* MLST. First, we identified the most variable genes with accumulated single nucleotide variants (SNVs) in short DNA fragments (genes containing the highest SNVs frequency per kbp) and, at the same time, with potential ability to distinguish all strains used for this analysis (containing 22 and more variable sites; Table S1). We identified six candidate genes (Table 1) and compared the resolution power of phylogenetic trees based on genome-wide data and phylogenetic trees based on sequences of individual genes.

**Table 1:** Genes with the highest SNVs frequency per kbp containing 22 and more SNVs among samples listed in Table S1.

Locus^$^	Length	Protein function	No. of variable sites	SNVs frequency/kbp
*TPESAMD_0136*	1,412	fibronectin-binding protein	41	29.04
*TPESAMD_0548*	1,298	FadL ortholog, outer membrane protein	36	27.73
*TPESAMD_0858*	1,229	FadL ortholog	33	26.85
*TPESAMD_0488*	2,537	methyl-accepting chemotaxis protein	31	12.22
*TPESAMD_0865*	1,445	FadL ortholog	26	17.99
*TPESAMD_0326*	2,502	BamA	22	8.40

^$^Anotation and length of the genes were identified according to the *TPE* reference genome Samoa D (GenBank accession number CP002374.1). Protein predictions by Brinkman *et al*.^35^ and Radolf and Kumar^36^.

With this approach, we propose a new MLST scheme for *TPE* strains of NHP origin, based on sequencing of two variable loci (*TP0548* and *TP0488*). The typing scheme is able to reveal 70% of whole genome resolution. Further details on the identification of most variable genes, resolution power, and the selection of most suitable typing loci are provided in the Technical Appendix.

### Samples included into the study and DNA extraction

Our study used *TP* positive DNA samples from 85 NHPs of six different species and three African countries (Tables 2 and S3). The samples originated from previously published^2,13^ and ongoing research investigations. The different methods of DNA extraction are presented in the Technical Appendix.

**Table 2:** Overview of NHP species, sample types, and geographic origin.

NHP species	Sample type (n NHPs)^$^	Geographic origin	Reference
Olive baboon (*Papio anubis*)	Skin (61)^%^, lesion swab (3)	Tanzania	(2)
Yellow baboon (*Papio cynocephalus*)	Skin (7)	Tanzania	(2)
Vervet monkey (*Chlorocebus pygerythrus*)	Skin (8)	Tanzania	(2)
Blue monkey (*Cercopithecus mitis*)	Skin (1)	Tanzania	(2)
Grivet monkey (*Chlorocebus aethiops*)	Lesion swabs (2)	Ethiopia	Ongoing research, unpublished
Western lowland gorilla (*Gorilla gorilla gorilla*)	Feces (4)	Republic of the Congo	Ongoing research, unpublished

Only previously *TP* positive tested samples have been included. ^$^The number of NHPs (n) which were sampled is not necessarily equal to the number of strain sequences. In a few cases (n = 3) multi-strain infection was present, which increased the sequence data output. ^%^Including one lymph node aspiration sample.

### DNA target enrichment

Before MLST, DNA extracted from fecal samples was enriched for bacterial DNA using the Looxter Enrichment Kit (Analytik Jena, Jena, Germany) following the manufacturer’s protocol.

### Polymerase chain reactions

#### Multi-Locus Sequence Typing system

*TP0548*: Amplification of a fragment of the *TP0548* gene was achieved using a nested PCR. The two-step PCR amplified a 1,065-bp long fragment of the target gene. Amplification and sequencing primers were used as reported elsewhere^14^. Briefly, the 50-µl reaction volume comprised 25 µl of the 2x Universe buffer (Universe High Fidelity Hot Start DNA Polymerase Kit, Biotool, Munich, Germany), 17 µl RNAase free water, 2 µl of each 10 µM primer, 1 µl DNA polymerase (1 U/µl), 1 µl of the dNTP mix (10 mM each), and 2 µl template DNA, independent of DNA concentration. Amplification was performed in a SensoQuest Labcycler using the following thermocycler conditions: pre-denaturation at 95 °C for 3 min, followed by 40 and 30 cycles, respectively, each with 95 °C for 15 sec, 48 °C for 15 sec, and 72 °C for 30 sec. Each of the PCR runs ended with a post-extension step at 72 °C for 5 min.

*TP0488*: The PCR amplified a 837 bp-long fragment of the *TP0488* gene using 5′-CCC TGC GCA CCA AGC TC-3′ and 5′-ACA CAG GCC CCA TAA ACT-3′ primers. Briefly, the 51-µl reaction volume comprised 45 µl Platinum PCR Super Mix High Fidelity (ThermoFisher Scientific, Munich, Germany), 2 µl of each 10 µmol/L primer, and 2 µl template DNA, independent of DNA concentration. Amplification was performed in a SensoQuest Labcycler using the following thermocycler conditions: pre-denaturation at 94 °C for 2 min, followed by 80 cycles each with 94 °C for 15 sec, 59 °C for 15 sec, and 68 °C for 1 min.

#### Additional gene targets

*TP0619*: Although the *TP0619* locus is not part of the newly designed typing system, it was amplified to further discriminate between *TPA* and *Treponema pallidum* subsp. *endemicum* (*TEN*) strains. We performed the PCR as described previously^2^. PCR conditions included a pre-denaturation at 95 °C for 3 min, followed by 40 cycles each with 95 °C for 15 sec, 55 °C for 15 sec, and 72 °C for 30 sec. The PCR run ended with a post-extension step at 72 °C for 5 min.

### Gel electrophoresis, purification, and DNA sequencing

All PCR products were run on 1% agarose gels to check for PCR performance and correct amplicon size. Products of the appropriate size were gel extracted and purified with the Qiagen Gel Extraction Kit (Qiagen, Hilden, Germany) and subsequently Sanger sequenced using the Microsynth SeqLab Laboratory service (Microsynth, Göttingen, Germany).

### 23S ribosomal RNA gene restriction enzyme analysis

Additional analyses of the 23 S ribosomal RNA gene was conducted to identify the two point-mutations including A2058G and A2059G that encode for macrolide resistance^14–16^. We note here that this locus is not part of the MLST, but it provides an essential information on macrolide resistance in NHP infecting *TP* strains. Methods were performed according to the procedures described by Lukehart *et al*.^17^ with minor modifications. Amplification was done using a semi-nested PCR. The primers of the first PCR were S-primer 5′-GTA CCG CAA ACC GAC ACA G-3′ and AS-primer 5′-GCG CGA ACA CCT CTT TTT AC-3′ using an annealing temperature of 62 °C and 35 cycles. All other PCR conditions were identical with those for *TP0619*. The second PCR was performed with the S-primer from the first PCR and AS-primer 5′-AGT CAA ACC GCC CAC CTA C-3′. Amplification was conducted with an annealing temperature of 63 °C and 30 cycles. Again, all other PCR conditions were the same as for *TP0619*. PCR products were run on 1% agarose gels to check for the expected 628 bp-sized PCR product. Subsequently, each sample was digested overnight with restriction enzyme *Mbo* II (R0148, BioLabs New England, Beverly, MA, USA) at 37 °C and in a second reaction with *Bsa* I (R0535, BioLabs New England, Beverly, MA, USA) at 50 °C. Interpretation of results was performed as published previously^17^. *TPA* strains Street 14 (known mutation at A2058G, digested with *Mbo* II) and UW330B (known mutation at A2059G, digested with *Bsa* I) were included as positive controls.

### Data analysis

Sequence data were analyzed, edited, and aligned using Geneious v11.1.4 (Biomatters Ltd., Auckland, New Zealand). We compared sequences with respective orthologs available in GenBank using BLAST search (http://blast.ncbi.nlm.nih.gov/Blast.cgi) with megablast or blastn tools. The Free Phylogenetic Network Software (www.fluxus-eneineering.com) was used to create a median-joining network as described elsewhere^18^. The respective concatenated sequence alignment (*TP0548* and *TP0488*) was used in combination with the assigned traits ‘NHP species’ and ‘sample location trait’. Maximum-parsimony (MP) trees, with gaps coded as fifth character, were constructed with SeaView 4.5.6^19^. Maximum-likelihood (ML) trees were calculated in IQ-TREE 1.6.1^20^ with the respective best-fit models. To obtain node support, all trees were constructed with 1,000 bootstrap replicates.

## Results

**Figure 1:**
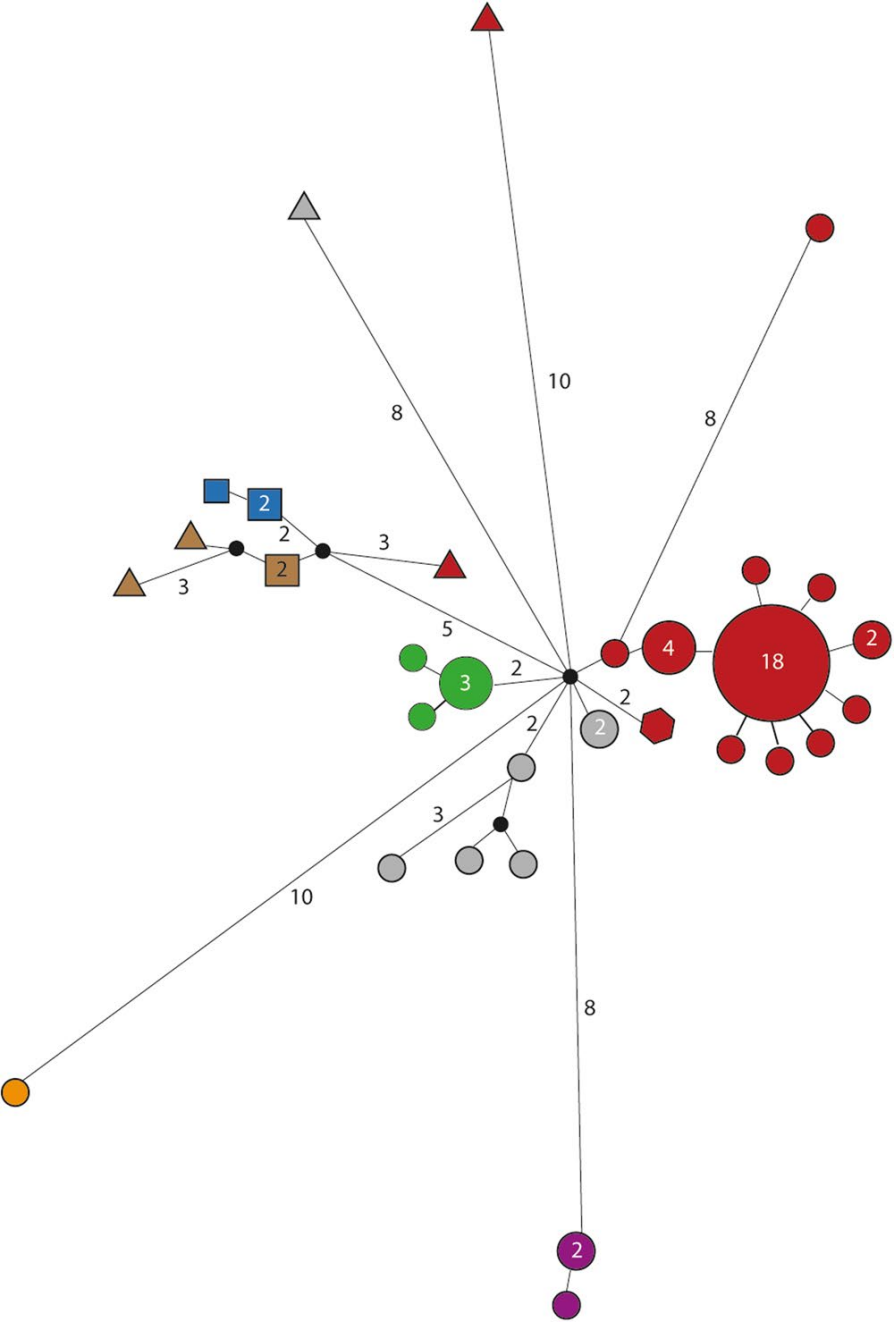
Median-joining network using 1,773 bp – long concatemer of *TP0488* and *TP0548* loci from 57 Tanzanian NHPs samples. The number of mutations, when >1, is given close to branches. Inferred allelic variants (median vectors) are shown as small black connecting circles. If contiguous, indels were considered as a single event only. The number of individuals, when >1, is shown inside the circles and are dependent on circle size. Species trait is given in the geometric form: circle = *Papio anubis* (n = 46); squares = *Papio cynocephalus* (n = 5); triangles = *Chlorocebus pygerythrus* (n = 5); hexagon = *Cercopithecus mitis* (n = 1). Sample location trait is given by the color code: blue = UG (n = 3); orange = TN (n = 1); brown = Ruaha National Park (n = 4); red = Lake Manyara National Park (n = 34); grey = Serengeti National Park (n = 7); green = Ngorongoro Conservation Area (n = 5); violet = Gombe National Park (n = 3).

**Figure 2:**
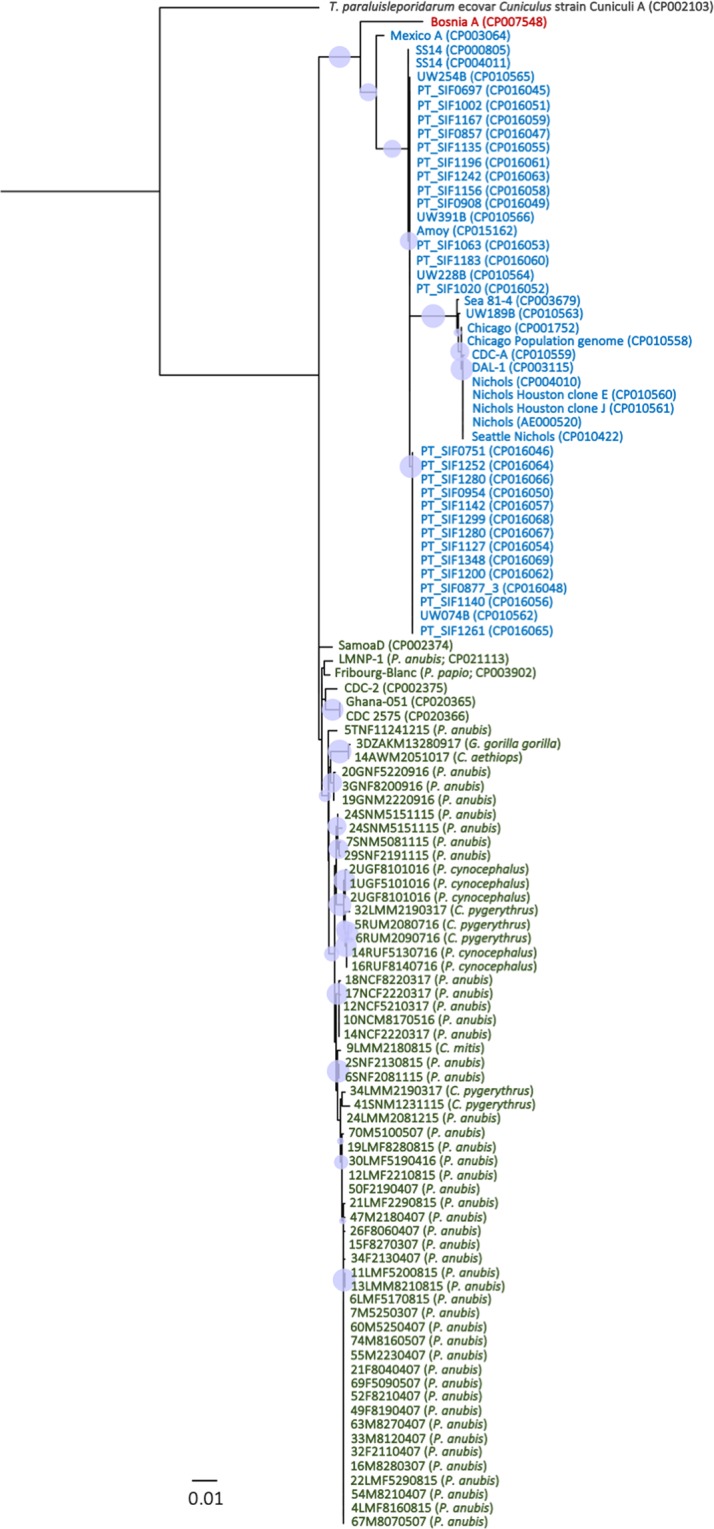
Rooted ML tree based on the concatenated sequences used for MLST (*TP0548* and *TP0488*). The tree is based on 1,773 nts and 1,000 bootstrap replicates. Bootstrap values from 80–100% are highlighted as light blue circles of respective size. NHP species and/or GenBank accession numbers of published strains are provided in parentheses following the name of the strain. In all cases were the species is not mentioned, sequences are from *TP* of human origin. Blue = subsp. *pallidum*, green = subsp. *pertenue*, red = subsp. *endemicum*. The pathogen causing rabbit syphilis, *Treponema paraluisleporidarum* ecovar *Cuniculus* strain Cuniculi A, is used as an outgroup. The bar refers to substitutions per site.

PCR results obtained from 85 NHP samples, of which 79 originated from Tanzanian monkeys (olive baboon, yellow baboon, vervet monkey, and blue monkey), two from Ethiopian grivet monkeys (*Chlorocebus aethiops*), and four from western lowland gorillas (*Gorilla gorilla gorilla*) from the Republic of the Congo, are summarized in Table S3. In general, PCR performances of the two gene targets used for the strain typing were equivalent with 78 (*TP0548*) and 67 (*TP0488*) resulting sequence data. This created a total of 59 concatenated sequences (the two gene targets combined) that were used for further analysis. We excluded the grivet monkey and the gorilla sample from the network analysis (Fig. 1) in order to maintain the highest possible resolution for the Tanzanian NHP samples where a high number of samples came from one geographic location. However, we used the complete concatenated sequence alignment, including the data obtained from the grivet monkey and gorilla samples, to construct ML (Fig. 2) and MP (Fig. S6) trees. For the latter, gaps were coded as fifth character. NHP strains included in this study clustered with human yaws-causing strains and were clearly separated from the *TEN* strain Bosnia A as well as the human syphilis-causing *TPA* strains (Figs 2 and S7). Within the *TPE* clade, bootstrap support was mostly weak (<80%) with some exceptions. One of these notable exceptions was the separation of the grivet monkey infecting strain from Ethiopia and the strain that was generated from a gorilla fecal sample. Sequences of both strains (14AWM2051017 and 3DZAKM13280917) differed in only one nucleotide position and always clustered together (Figs 2 and S6). Overall, we found a geographic clustering of *TPE* strains, instead of clustering by host species. None of the samples from the Tanzanian NHPs (n = 72/76; four samples did not generate a PCR product) has been tested positive for the mutations in the 23 S ribosomal RNA genes that code for macrolide resistance. The grivet monkey and the gorilla samples were not tested for microbial resistance.

Sequences obtained from the *TP0619* gene were identical across all NHP species and sampling sites, including the grivet monkey and the gorilla samples. A representative sequence obtained from a vervet monkey sampled at Katavi National Park (4KNF2121016) was published previously under the GenBank accession number MF754122^2^.

In general, all NHP-derived sequences of the *TP0548* locus including the grivet monkey and the gorilla sequence, contained one section of the sequence where most of the nucleotide variation is found (Fig. S1). This distinguishes them not only from human syphilis- (*TPA*) and bejel-causing (*TEN*) strains but also from *TPE* strains of human origin, where there are three and two variable regions, respectively. Sequences of the *TP0548* and *TP0488* loci showed comprehensive variability within and across the different sampling locations as well as between the different NHP species in Tanzania. Corresponding ML and MP trees each constructed for *TP0548* (Figs S2 and S3) and *TP0488* (Figs S4 and S5) were similar in topology. We note here that sample size was low for some of the species (e.g., blue monkey) and that we included here only the Tanzanian samples to match the network analysis (Fig. 1).

**Figure 3:**
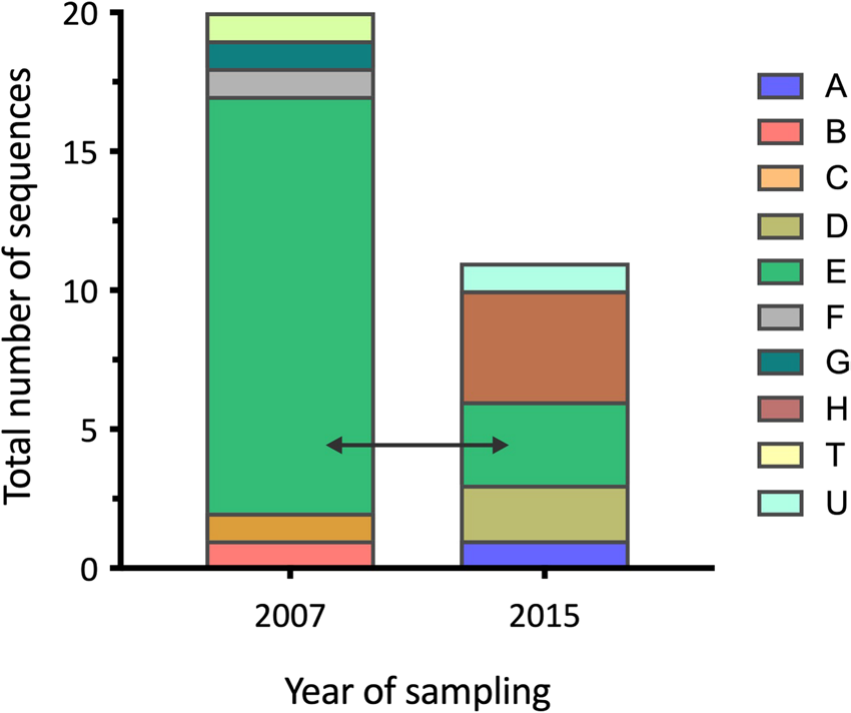
Temporal strain composition for samples collected from olive baboons at Lake Manyara National Park. The different colors indicate different genotypes. Sampling of olive baboons at LMNP was conducted in 2007 and 2015. The strain “E” (light green; e.g., 4LMF8160815) was the dominating strain in 2007 and was still present in baboons sampled eight years later (2015).

Since the largest number of samples was taken at Lake Manyara National Park (LMNP) and samples were collected in 2007, 2015, and 2017, respectively, we were able to plot the temporal strain composition for this specific sampling location. Figure 3 illustrates the temporal strain composition for all samples that originate from olive baboons. Using a sequence alignment and base-by-base comparison, we found that in 2007 there were six strains, with a dominating strain “E” (e.g., 4LMF8160815). Samples from olive baboons at LMNP taken eight years later reconfirmed the existence of this strain. The temporal stability of strains was further supported by strain “P” (e.g., 10NCM8170516) that was collected from two olive baboons at the Ngorongoro Conservation Area in 2015 and one olive baboon in 2016 (Table S4).

## Discussion

Frequent transmission of *TPE* strains across the different NHP species would likely result in a higher number of shared and identical sequences in different species at the same geographic site. Although we found no identical strain sequences in different primate species at one site (Table S4), we still observed a geographic clustering of (closely related) *TPE* strain sequences and not a clustering according to NHP host species (Fig. 1). This suggests that interspecies transmission occurs, albeit rarely. That we found no identical strain sequences in different primate species could be due to the small sample size for some of the investigated NHP species, but generally argues against frequent transmission. This gains further support by our finding that the strains of NHP origin are relatively stable over time as we show for LMNP (Fig. 3, Table S4). This is consistent with what we know from human infecting *TPA* strains^21–23^. The 2007 dominating strain “E” (e.g., 4LMF8160815), which was isolated from olive baboons was still present in the infected baboon population in 2015. Although we do not see a strict geographic pattern when the two target genes were analyzed individually (Figs S2–S5), when examining the concatenated alignment (median-joining network (Fig. 1) and the ML tree (Fig. 2)), geographic strain clustering can be observed. This is another indicator for the relative temporal and geographic stability of the strains that infect NHPs. The recently estimated low mutation rate in human *TPE* strains^22^ coincides with the genetic stability observed among NHP *TPE* strains analyzed in this study. Yet, feasible interspecies transmission routes for *TP* exist^3^ and have been discussed for flies^24^ and were proven for sexual intercourse between different NHP species based on host genetic data^25^.

Despite the differences in the number of variable sites at the *TP0548* locus (Fig. S1), we see a close association of NHP and human infecting *TPE* strains. This was expected, since the genome of *TPE* LMNP-1 strain (GenBank accession number CP021113), which was obtained from an olive baboon at LMNP in 2007, was found to be closely related to human yaws causing strains similar to all other *TPE* strains of NHP origin^1^.

The absence of antibiotic resistance to azithromycin in all tested *TP* strains from NHPs in Tanzania is a positive sign and is probably related to the absence of treatment of infected monkeys. Currently only Gombe National Park has a history of treating infected baboons with antimicrobials^26^. In human yaws, it has been shown that after a single treatment round with antibiotic macrolides, resistance emerges^27^ even though the *de novo* emergence of such mutations is lower than 10^−3^ per treated patient^28^. The risk of emerging antimicrobial resistance is of major concern for human infection and would also draw major implications for the conservation of endangered NHP species such as gorillas (*Gorilla gorilla*). Similar to human yaws elimination^27^, responsible treatment of infected NHP populations requires resistance monitoring and possible ring-fencing with effective alternative antimicrobials.

The identification of two different strains obtained from independent PCRs in three different NHPs (*TP0548*: 11LMF8190815 (*P*. *anubis*; 15-bp indel); *TP0488*: 2UGF8101016 (*P*. *cynocephalus*; one SNV) and 24SNM5151115 (*P*. *anubis*; three SNVs); Table S4) supports the concept of absence of cross immunity between different *TP* strains^29^ and boosts evidence for recombination events found in the *TP* bacterium^30–32^ under natural conditions.

The initial CDC typing system for *TPA* made use of the number of 60-bp repeats found in the acidic repeat protein (*arp* (*TP0433*)) gene in combination with differences found in the *tpr* subfamily II genes (*tprE* (*TP0313*), *tprG* (*TP0317*), and *tprJ* (*TP0621*)^9^. A subsequently introduced enhanced typing system included a portion of the *TP0548* locus^33^. While the enhanced typing system has also been used for the typing of human *TPE* strains^12^, it did not overcome the difficulties associated with amplification of 60-bp repeats of the *arp* gene or the uncertainties associated with amplification of three different *tpr*-subfamily II genes in one single assay, followed by subsequent restriction enzyme analysis^11^. A recently published alternative method for MLST included the widely used *TP0548* locus but also two additional loci located in the *TP0136* and *TP0326* genes^11^. We took these loci into account, but in our analysis, it became evident that the *TP0136* locus in the Tanzanian *TP* strains of NHP origin was highly conserved. A representative sequence is accessible under GenBank accession number CP021113.1 (nt158,275-159,195). Whether this is a characteristic of NHP infecting strains or a spatial property of strains originating from Tanzania is unknown. It underlines, however, that a globally applied strain typing system for *TPE* requires a comprehensive database of high-quality genomes obtained from larger numbers of clinical samples from yaws endemic areas.

We identified several gene loci as suitable candidates to be used in *TPE* strains of human and NHP origin. The two gene targets that we selected for MLST (*TP0548* and *TP0488*) originated from a number of suitable candidates (Table 2) and selection was based on best PCR performance in the clinical NHP samples that were included in this study. We neither aimed for the design of a *TPE* typing system that can be used in a clinical environment for human infection, nor did we anticipate a typing system that is suitable for a global approach. Amplification of relatively long sequence parts (e.g., *TP0548* enhanced typing system determines 84 bp^33^ vs. 1,065 bp determined in this study) was therefore not considered an issue. Both loci that were used for the typing, *TP0548* and *TP0488*, are reported to show signs of recombination in human syphilis causing *TPA* strains^30–32^. This, however, was not considered a limitation since our MLST typing system was designed to describe strain variability within a given population of NHPs and is not used to describe a detailed geophylogeny of *TP*. While the *TP0619* sequence, which codes for a protein family of Domains of Unknown Function (DUF)2715 and which appear to be restricted to *TP*, is not part of our typing system, it is useful to support the difference of the *TP* strains of NHP origin analyzed in this study from human syphilis-causing *TPA* strains. It is currently unclear if wild NHPs are also naturally infected with *TPA* strains.

Invasive sampling of NHPs in the wild is generally associated with challenges in terms of ethics and logistics. As a consequence, it is easier to obtain a greater number of non-invasively collected fecal samples than samples that originate from invasively sampled individuals. Moreover, prospective epidemiological studies would benefit from sampling regimes that allow the screening and subsequent typing of *TP* strains in non-invasively collected samples from a greater geographic area. For this reason, we tested our newly established MLST system in noninvasively collected fecal samples from gorillas at Odzala-Kokoua National Park in the Republic of the Congo, a place where gorillas with ulcerative skin lesions have been frequently sighted^34^ (Fig. S7). We were able to successfully strain type the gorilla samples and could show that the strain from which we obtained all three target sequences, clusters with a strain isolated from a grivet monkey in Ethiopia. The reason for the close association of the strain of the grivet monkey and the gorilla origin is currently unclear and answering this question requires intensified sampling in the respective geographic areas and in-between to obtain more sequence data.

## Conclusion

The high number of *TP* infected NHPs in Africa, the different species that have been confirmed as hosts^2,4^, as well as the recently documented close genetic and functional similarity of NHP and human infecting *TPE*^1^ requires epidemiological data for a better understanding on how the infection is maintained in primate populations and whether or not it is transmitted to humans. Our study provides an important contribution to answer the question on interspecies transmission in primate infecting *TP*, although further sampling is needed to increase confidence in the results. However, with our data we were able to show that interspecies transmission in Tanzanian monkeys is likely although rare. As humans are primates, the most important question to answer in future studies is, whether or not *TPE* strains of NHP origin transmit to humans and/or *vice versa*. The interspecies transmission of *TPE* in nonhuman primates, is not necessarily predictive for spillovers to humans.

## Supplementary information

Technical appendix

Table S5

## Data Availability

GenBank accession numbers for the sequences generated in this study can be found in Table S5.
