# Supplementary material for: Strain diversity of Treponema pallidum subsp. pertenue suggests rare interspecies transmission in African nonhuman primates
Source: Sci Rep. 2019 Oct 2;9:14243. doi: 10.1038/s41598-019-50779-9 (PMC6775232; doi:10.1038/s41598-019-50779-9)
Supplement: Supplementary file 1 — Technical appendix [file 41598_2019_50779_MOESM1_ESM.pdf]

Technical appendix

Strain diversity of *Treponema pallidum* subsp. *pertenue* suggests rare interspecies transmission in African nonhuman primates

Authors: Idrissa S. Chuma[1,2,3*], Christian Roos[2,4*], Anagaw Atickem[5], Torsten Bohm[6], D. Anthony Collins[7], Linda Grillová[8,9], Luisa K. Hallmaier-Wacker[1,2], Rudovick R. Kazwala[3], Julius D. Keyyu[9], Simone Lüert[1,2], Ulrich Maloueki[6,11], Jan Oppelt[9,12], Klára J. Petrželková[13,14,15], Alexander Piel[16,17], Fiona A. Stewart[16,17], David Šmajs[9], Sascha Knauf[1,18§]

Design of the Multi-Locus Sequence Typing system

Identification of most variable genes in TPE

We identified six candidate genes with the SNVs frequency per kbp ranging from 8-29, which code outer membrane proteins or hypothetical proteins (*TP0136*, *TP0326*, *TP0488*, *TP0548*, *TP0858*, *TP0865*; Table 1). Most of the loci were identified as putative intra- or inter-strain recombination genes (*TP0136*, *TP0326*, *TP0488*, *TP548* and *TP0865*)[30,32,37-38].

Resolution power of genome-wide data and individual loci

Genome-wide maximum-likelihood (ML) trees of 23 available complete and draft genome sequences were based on 1,207 variable sites and allowed to distinguish 22 haplotypes (data not showed). Given the fact that strains CDC 2575 and Ghana-051 are completely identical[20], the genome-wide tree had 100% resolution of whole genome sequences. Among six candidate loci, the highest resolution power was observed in *TP0488* (70%) followed by *TP0326*,

TP0548, *TP0858* (all 57%), *TP0136* (44%) and *TP0865* (31.8%). Interestingly, the concatenated sequences of all candidate loci did not reveal higher resolution then the resolution observed in the *TP0488* gene (70%).

Identification of the most suitable typing loci for TPE MLST among and within the candidate genes

Since the amplification efficiency of *Treponema* DNA from clinical samples has been shown to be dependent on the length of the PCR products[10,39], we selected loci with the highest occurrence of variable sites accumulated in as short DNA regions as possible (Table S2). Furthermore, we preferred loci with the highest percentage of genome-wide resolution and last, we selected loci that were able to clearly distinguish treponematoses caused by *TPE* from the *TPA*/*TEN* infections (Table S2).

DNA extraction

DNA extraction for tissue samples was performed as described elsewhere[2]. Swab materials followed the same protocol as described for the skin tissues using the QIAamp DNA Mini Kit (Qiagen, Hilden, Germany) with some modifications. Briefly, each swab was digested in 450 µl custom-made lysis buffer (10 mM Tris [pH 8.0], 0.1 M EDTA [pH 8.0], and 0.5% sodium dodecyl sulfate), the same buffer in which the swab was collected. In addition, the remaining lysis buffer was also digested. In both reactions, care was taken to keep the ratio of buffer and proteinase K as recommended by the manufacturer. The samples were digested overnight at 56°C and 900 rpm (Thermomixer Comfort, Eppendorf, Hamburg, Germany). On the next day, reaction tubes that contained swabs were placed on top of a new PCR clean reaction tube of 2.0 ml volume. A sterile 20G needle was used to penetrate the bottom of the reaction tube to allow for subsequent separation of DNA containing fluid from the swab through vigorous centrifugation at 6,000 xg for 5 min at room temperature. All subsequent steps followed the

manufactures protocol. The DNA was eluted twice with 100 µL AE buffer and was further purified using glycogen precipitation according to the protocol published in Knauf et al.[24].

Ethanol (98%)-preserved fecal samples from western lowland gorillas (*Gorilla gorilla gorilla*) were extracted using the First DNA All-tissue extraction kit (Gen-ial, Troisdorf, Germany). Briefly, the procedure followed the manufacturer's guidance with some minor modifications. Feces were dried from ethanol overnight and subsequently resolved in 1 ml of the kit's containing lysis buffers #1 and 100 µl of lysis buffer #2. Samples where then incubated with 20 µl proteinase K at 65°C and 1000 rpm (Thermomixer Comfort, Eppendorf, Hamburg, Germany) for 50 min. All subsequent steps followed those of the manufacture's protocol.

Table S1. *TPE* genomes used to identify most suitable gene candidates for the MLST design. [$] In order to determine the whole genome sequence, the *TPE* strain was multiplied in experimental animals prior to the NGS. [§] Culture-independent enrichments (hybridization captures) were used prior to the NGS in order to separate the *TP* genetic material from the host DNA. Compl.=complete

Strains ID	Genome	Source	Host	Year of isolation	Geographic area	References
CDC-1	Compl.	Rabbit inoculation[$]	Human	1980	Ghana	unpublished
CDC-2	Compl.	Rabbit inoculation[$]	Human	1980	Ghana	40
Samoa D	Compl.	Rabbit inoculation[$]	Human	1953	Western Samoa	40
Gauthier	Compl.	Rabbit inoculation[$]	Human	1960	Congo	40
Fribourg-Blanc	Compl.	Rabbit inoculation[$]	NHPs	1966	Ghana	41
CDC 2575	Compl.	Rabbit inoculation[$]	Human	1980	Ghana	21
Ghana-051	Compl.	Rabbit inoculation[$]	Human	1988	Ghana	21
Sei Geringging K403	Compl.	Rabbit inoculation[$]	Human	1990	Indonesia	42
Kampung Dalan K363	Compl.	Rabbit inoculation[$]	Human	1990	Indonesia	42
LMNP-1	Compl.	Clinical[§]	NHPs	2007	Tanzania	1
LMNP-2	Draft	Clinical[§]	NHPs	2007	Tanzania	1
Gambia-1	Draft	Clinical[§]	NHPs	unknown	Gambia	1
Gambia-2	Draft	Clinical[§]	NHPs	unknown	Gambia	1
Senegal NKNP-1	Draft	Clinical[§]	NHPs	unknown	Senegal	1
Senegal NKNP-2	Draft	Clinical[§]	NHPs	unknown	Senegal	1
Cote d'Ivoire TaiNP-1	Draft	Clinical[§]	NHP	unknown	Ivory Coast	1
Cote d'Ivoire TaiNP-2	Draft	Clinical[§]	NHP	unknown	Ivory Coast	1

ERR1470330	Draft	Clinical[§]	Human	2013	Solomon Islands	43
ERR1470331	Draft	Clinical[§]	Human	2013	Solomon Islands	43
ERR1470334	Draft	Clinical[§]	Human	2013	Solomon Islands	43
ERR1470338	Draft	Clinical[§]	Human	2013	Solomon Islands	43
ERR1470343	Draft	Clinical[§]	Human	2013	Solomon Islands	43
ERR1470344	Draft	Clinical[§]	Human	2013	Solomon Islands	43

Table S2: Characteristics of candidate genes for *TPE* typing. $ According to the yaws reference genome Samoa D (GenBank accession number CP002374.1). § The genomes listed in Table 1 including representatives of *TPE* strains, representative of TEN (Bosnia A, GenBank accession number CP007548.1) and representatives of *TPA* (Nichols, GenBank accession number CP004010.2; SS14, GenBank accession number CP004011.1; Mexico, GenBank accession number CP003064.1) were used to test whether the corresponding sequences can be used for subspecies classification (*TPA*/*TPE*/*TEN*).

Gene$	Variable region length (bp)	Variable region coordinates$	% of genome-wide data resolution	Differen-tiating TPE from TPA/TEN§	Differen-tiating TPA/TPE/TEN§
TP0488	782	522,942 – 523,723	70.0	Yes	No
TP0548	755	593,318 – 594,072	57.0	No	No
TP0858	824	936,118 – 936,941	57.0	Yes	Yes
TP0326	2,086	346,066 – 348,151	57.0	Yes	No
TP0136	910	157,823 – 158,733	44.0	Yes	Yes
TP0865	897	945,224 – 946,121	31.8	Yes	Yes

Table S3. Summary of *TP* sequence data of NHP origin included into this study. *multi-strain infection present, NP=National Park, CA=Conservation Area, TZ=Tanzania, ET=Ethiopia, RC=Republic of the Congo. Details on NHP species composition can be found in Table S5.

Country	Sample location	n NHPs	n *TP0619* sequences	n *TP0548* sequences	n *TP0488* sequences	n concatenated sequences
TZ	Gombe NP	3	1	3	3	3
TZ	Lake Manyara NP	46	41	44*	37	33
TZ	Katavi NP	1	1	0	1	0
TZ	Mahale NP	1	1	1	0	0
TZ	Mikumi NP	1	0	1	0	0
TZ	Ngorongoro CA	9	9	9	5	5
TZ	Ruaha NP	6	4	5	5	4
TZ	Serengeti NP	7	5	7	8*	8
TZ	Tarangire NP	2	1	2	1	1
TZ	Issa Valley	2	2	2	3*	3
TZ	Udzungwa NP	1	1	1	0	0
ET	Awash NP	2	1	2	1	1
RC	Odzala-Kokoua NP	4	4	1	4	1
	Total	85	71	78	67	59

Table S4. Strains diversity and year of sampling based on the concatenated sequences used for MLST (*TP0548* and *TP0488*) in NHP infecting *TP*. Strain classification letters (uppercase) shall not be confused with lower case letters that are used in the enhanced typing system in human syphilis and yaws-causing strains. The letters used here are only used to visualize and discuss the differences in *TP* strains of NHP origin.

Strain Typ	NHP sample ID	Sampling Area	Sampling Year	Species
A	21LMF2290815	LMNP	2015	*Papio anubis*
B	26F8060407	LMNP	2007	*Papio anubis*
C	47M2180407	LMNP	2007	*Papio anubis*
D	11LMF5200815	LMNP	2015	*Papio anubis*
D	13LMM8210815	LMNP	2015	*Papio anubis*
E	4LMF8160815	LMNP	2015	*Papio anubis*
E	6LMF5170815	LMNP	2015	*Papio anubis*
E	7F5250307	LMNP	2007	*Papio anubis*
E	15F8270307	LMNP	2007	*Papio anubis*
E	16M8280307	LMNP	2007	*Papio anubis*
E	21F8040407	LMNP	2007	*Papio anubis*
E	22LMF5290815	LMNP	2015	*Papio anubis*
E	32F2110407	LMNP	2007	*Papio anubis*
E	33M8120407	LMNP	2007	*Papio anubis*
E	49F8190407	LMNP	2007	*Papio anubis*
E	52F8210407	LMNP	2007	*Papio anubis*
E	54M8210407	LMNP	2007	*Papio anubis*
E	55M2230407	LMNP	2007	*Papio anubis*
E	60M5250407	LMNP	2007	*Papio anubis*
E	63M8270407	LMNP	2007	*Papio anubis*
E	67M8000507	LMNP	2007	*Papio anubis*
E	69F5090507	LMNP	2007	*Papio anubis*
E	74M8160507	LMNP	2007	*Papio anubis*
F	34F2130407	LMNP	2007	*Papio anubis*
G	70M5100507	LMNP	2007	*Papio anubis*
H	12LMF2210815	LMNP	2015	*Papio anubis*
H	19LMF8280815	LMNP	2015	*Papio anubis*
H	30LMF5190416	LMNP	2015	*Papio anubis*
H	50F2190407	LMNP	2015	*Papio anubis*
I	5TNF11241215	TNP	2015	*Papio anubis*
J	24SNM5151115	SNP	2015	*Papio anubis*
K	29SNF2191115	SNP	2015	*Papio anubis*
L	7SNM5081115	SNP	2015	*Papio anubis*

M	24SNM5151115	SNP	2015	*Papio anubis*
N	20GNF5220916	GNP	2016	*Papio anubis*
O	19GNM2220916	GNP	2016	*Papio anubis*
O	3GNF8200916	GNP	2016	*Papio anubis*
P	10NCM8170516	NCA	2016	*Papio anubis*
P	12NCF5210317	NCA	2017	*Papio anubis*
P	14NCM5210317	NCA	2017	*Papio anubis*
Q	17NCF2220317	NCA	2017	*Papio anubis*
R	18NCF8220317	NCA	2017	*Papio anubis*
S	2SNF2130815	SNP	2015	*Papio anubis*
S	6SNF2081115	SNP	2015	*Papio anubis*
T	LMNP-1	LMNP	2007	*Papio anubis*
U	24LMM2081215	LMNP	2015	*Papio anubis*
V	34LMM2190317	LMNP	2017	*Chlorocebus pygerythrus*
W	41SNM1231115	SNP	2015	*Chlorocebus pygerythrus*
X	9LMM2180815	LMNP	2015	*Cercopithecus mitis*
Y	5RUM2080716	RNP	2016	*Chlorocebus pygerythrus*
Z	14RUF5130716	RNP	2016	*Papio cynocephalus*
Z	16RUF8140716	RNP	2016	*Papio cynocephalus*
AA	6RUM2090716	RNP	2016	*Chlorocebus pygerythrus*
AB	32LMM2190317	LMNP	2017	*Chlorocebus pygerythrus*
AC	1UGF5101016	Issa Valley	2016	*Papio cynocephalus*
AC	2UGF8101016	Issa Valley	2016	*Papio cynocephalus*
AD	2UGF8101016	Issa Valley	2016	*Papio cynocephalus*

Table S5. GenBank accession numbers for the sequences included into this study. (Excel Sheet)

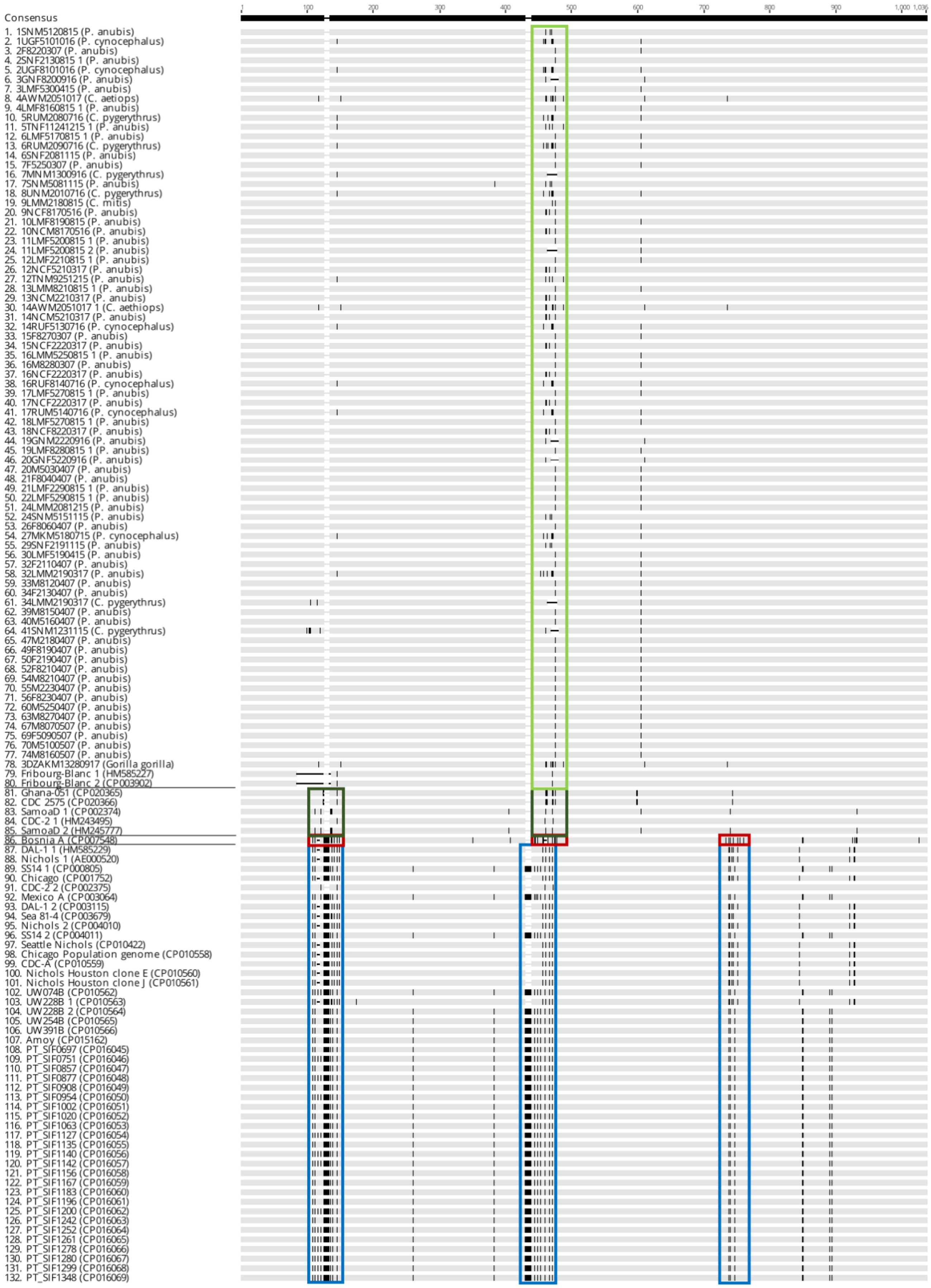

Consensus
1. 1SNM5120815 (P. anubis)
2. 1UGF5101016 (P. cynocephalus)
3. 2F8220307 (P. anubis)
4. 2SNF2130815 1 (P. anubis)
5. 2UGF8101016 (P. cynocephalus)
6. 3GNF8200916 (P. anubis)
7. 3LMF5300415 (P. anubis)
8. 4AWM2051017 (C. aetiops)
9. 4LMF8160815 1 (P. anubis)
10. 5RUM2080716 (C. pygerythrus)
11. 5TNF11241215 1 (P. anubis)
12. 6LMF5170815 1 (P. anubis)
13. 6RUM2090716 (C. pygerythrus)
14. 6SNF2081115 (P. anubis)
15. 7F5250307 (P. anubis)
16. 7MNM1300916 (C. pygerythrus)
17. 7SNM5081115 (P. anubis)
18. 8UNM2010716 (C. pygerythrus)
19. 9LMM2180815 (C. mitis)
20. 9NCF8170516 (P. anubis)
21. 10LMF8190815 (P. anubis)
22. 10NCM8170516 (P. anubis)
23. 11LMF5200815 1 (P. anubis)
24. 11LMF5200815 2 (P. anubis)
25. 12LMF2210815 1 (P. anubis)
26. 12NCF5210317 (P. anubis)
27. 12TNM9251215 (P. anubis)
28. 13LMM8210815 1 (P. anubis)
29. 13NCM2210317 (P. anubis)
30. 14AWM2051017 1 (C. aethiops)
31. 14NCM5210317 (P. anubis)
32. 14RUF5130716 (P. cynocephalus)
33. 15F8270307 (P. anubis)
34. 15NCF2220317 (P. anubis)
35. 16LMM5250815 1 (P. anubis)
36. 16M8280307 (P. anubis)
37. 16NCF2220317 (P. anubis)
38. 16RUF8140716 (P. cynocephalus)
39. 17LMF5270815 1 (P. anubis)
40. 17NCF2220317 (P. anubis)
41. 17RUM5140716 (P. cynocephalus)
42. 18LMF5270815 1 (P. anubis)
43. 18NCF8220317 (P. anubis)
44. 19GNM2220916 (P. anubis)
45. 19LMF8280815 1 (P. anubis)
46. 20GNF5220916 (P. anubis)
47. 20M5030407 (P. anubis)
48. 21F8040407 (P. anubis)
49. 21LMF2290815 1 (P. anubis)
50. 22LMF5290815 1 (P. anubis)
51. 24LMM2081215 (P. anubis)
52. 24SNM5151115 (P. anubis)
53. 26F8060407 (P. anubis)
54. 27MKM5180715 (P. cynocephalus)
55. 29SNF2191115 (P. anubis)
56. 30LMF5190415 (P. anubis)
57. 32F2110407 (P. anubis)
58. 32LMM2190317 (P. anubis)
59. 33M8120407 (P. anubis)
60. 34F2130407 (P. anubis)
61. 34LMM2190317 (C. pygerythrus)
62. 39M8150407 (P. anubis)
63. 40M5160407 (P. anubis)
64. 41SNM1231115 (C. pygerythrus)
65. 47M2180407 (P. anubis)
66. 49F8190407 (P. anubis)
67. 50F2190407 (P. anubis)
68. 52F8210407 (P. anubis)
69. 54M8210407 (P. anubis)
70. 55M2230407 (P. anubis)
71. 56F8230407 (P. anubis)
72. 60M5250407 (P. anubis)
73. 63M8270407 (P. anubis)
74. 67M8070507 (P. anubis)
75. 69F5090507 (P. anubis)
76. 70M5100507 (P. anubis)
77. 74M8160507 (P. anubis)
78. 3DZAKM13280917 (Gorilla gorilla)
79. Fribourg-Blanc 1 (HM585227)
80. Fribourg-Blanc 2 (CP003902)
81. Ghana-051 (CP020365)
82. CDC 2575 (CP020366)
83. SamoaD 1 (CP002374)
84. CDC-2 1 (HM243495)
85. SamoaD 2 (HM245777)
86. Bosnia A (CP007548)
87. DAL-1 1 (HM585229)
88. Nichols 1 (AE000520)
89. SS14 1 (CP000805)
90. Chicago (CP001752)
91. CDC-2 2 (CP002375)
92. Mexico A (CP003064)
93. DAL-1 2 (CP003115)
94. Sea 81-4 (CP003679)
95. Nichols 2 (CP004010)
96. SS14 2 (CP004011)
97. Seattle Nichols (CP010422)
98. Chicago Population genome (CP010558)
99. CDC-A (CP010559)
100. Nichols Houston clone E (CP010560)
101. Nichols Houston clone J (CP010561)
102. UW074B (CP010562)
103. UW228B 1 (CP010563)
104. UW228B 2 (CP010564)
105. UW254B (CP010565)
106. UW391B (CP010566)
107. Amoy (CP015162)
108. PT_SIF0697 (CP016045)
109. PT_SIF0751 (CP016046)
110. PT_SIF0857 (CP016047)
111. PT_SIF0877 (CP016048)
112. PT_SIF0908 (CP016049)
113. PT_SIF0954 (CP016050)
114. PT_SIF1002 (CP016051)
115. PT_SIF1020 (CP016052)
116. PT_SIF1063 (CP016053)
117. PT_SIF1127 (CP016054)
118. PT_SIF1135 (CP016055)
119. PT_SIF1140 (CP016056)
120. PT_SIF1142 (CP016057)
121. PT_SIF1156 (CP016058)
122. PT_SIF1167 (CP016059)
123. PT_SIF1183 (CP016060)
124. PT_SIF1196 (CP016061)
125. PT_SIF1200 (CP016062)
126. PT_SIF1242 (CP016063)
127. PT_SIF1252 (CP016064)
128. PT_SIF1261 (CP016065)
129. PT_SIF1278 (CP016066)
130. PT_SIF1280 (CP016067)
131. PT_SIF1299 (CP016068)
132. PT_SIF1348 (CP016069)
1
100
200
300
400
500
600
700
800
900
1,000 1,036

Figure S1. Alignment of NHP and human *TPA*, *TEN*, and *TPE* sequence data of *TP0548* locus. Sequences of *TP* of NHP origin show only one part of the sequence where most of the nucleotide variation is found (light green box). This distinguishes them from human *TPA* (blue box) and *TEN* (red box) as well as human yaws-causing strains (*TPE*; dark green box), where we have three and two variable regions, respectively. GenBank accession numbers for all NHP samples are listed in Table S5.

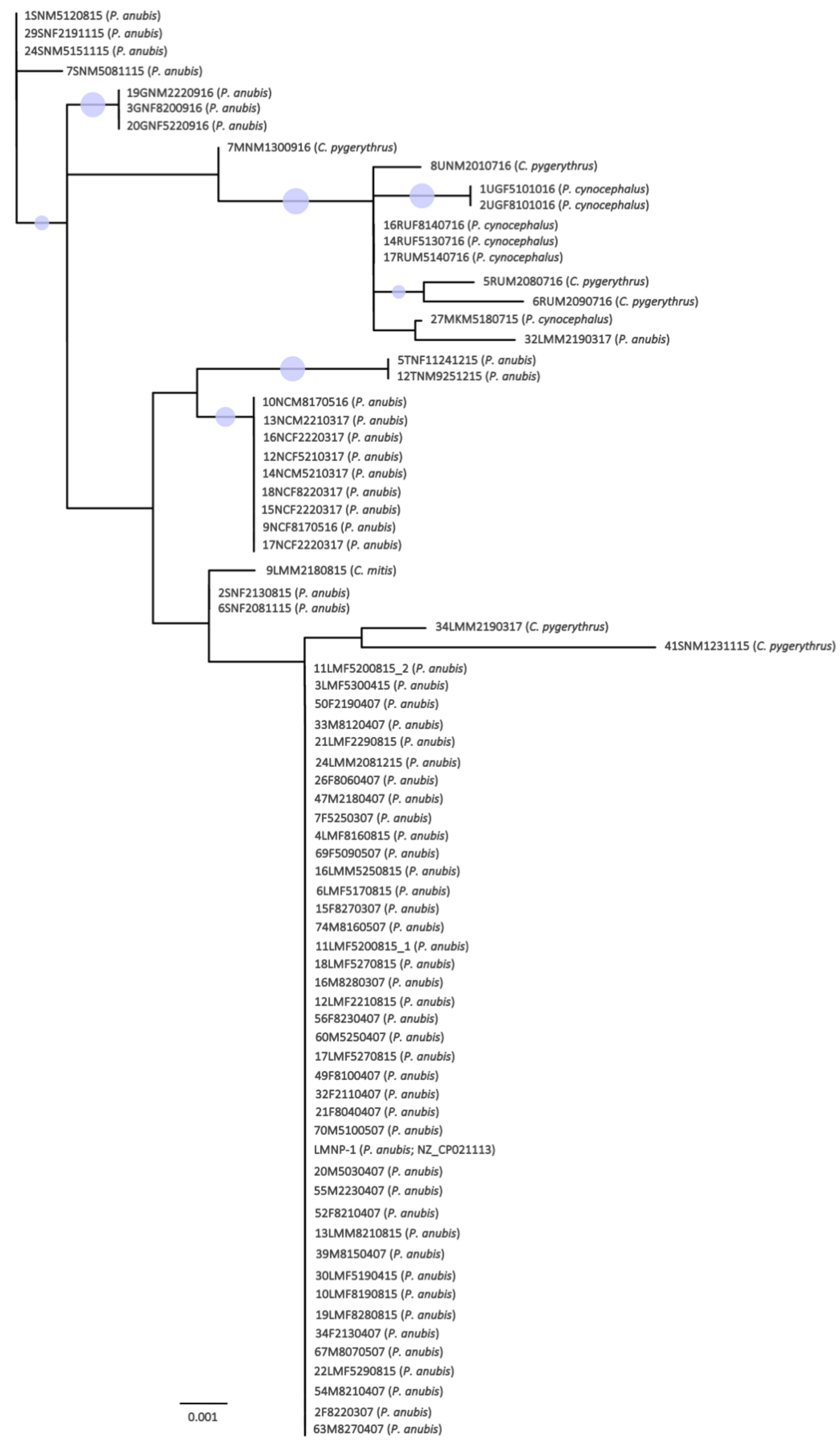

Figure S2. ML tree for the *TP0548* locus of the Tanzanian strains of NHP origin. 1,000 bootstrap replicates were performed and bootstrap values from 80-100% are highlighted as light blue circles of respective size. The bar indicates substitutions per site.

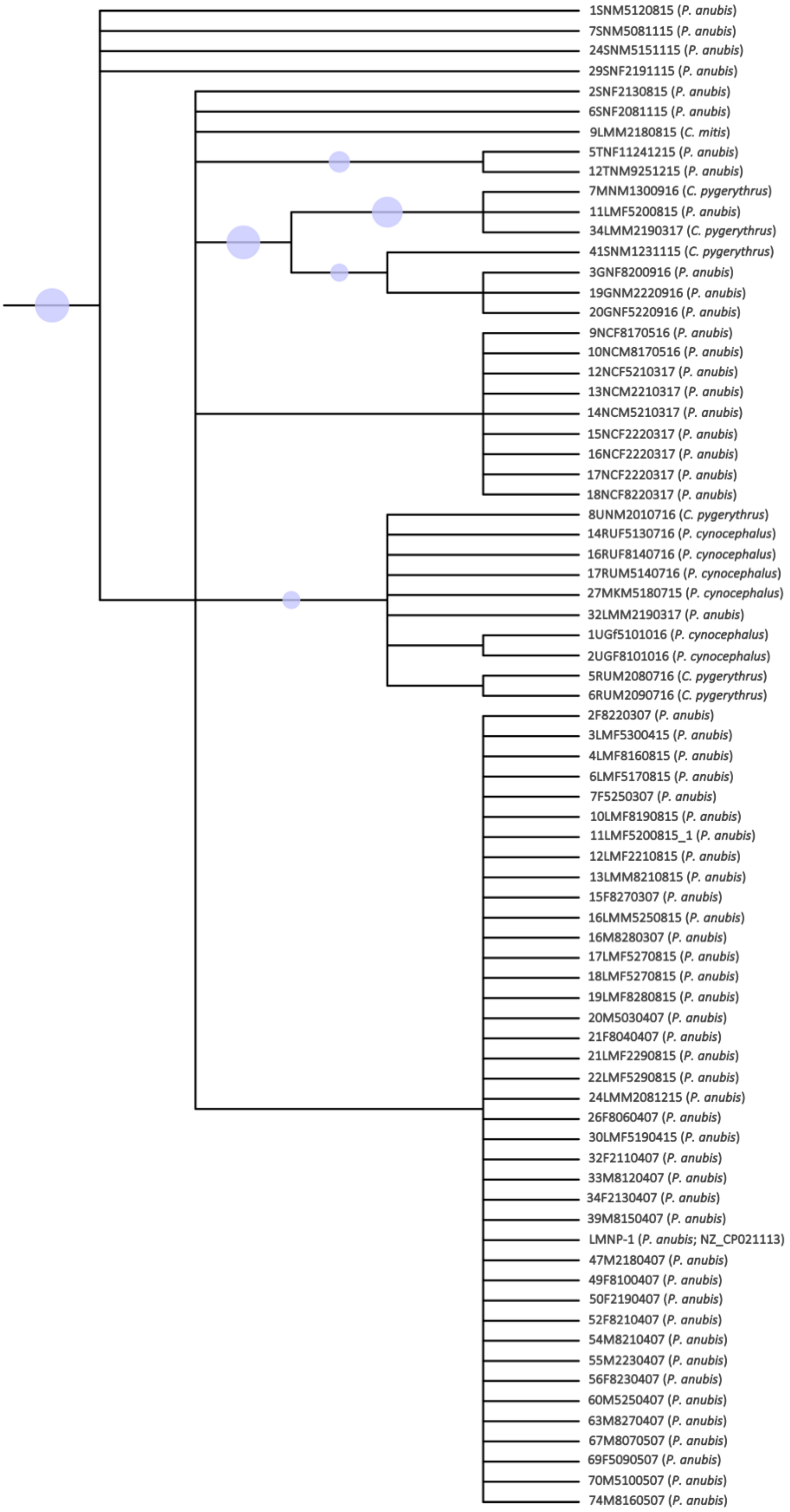

Figure S3. MP tree for the *TP0548* locus of the Tanzanian strains of NHP origin. 1,000 bootstrap replicates were performed and bootstrap values from 80-100% are highlighted as light blue circles of respective size. Gaps were coded as fifth character.

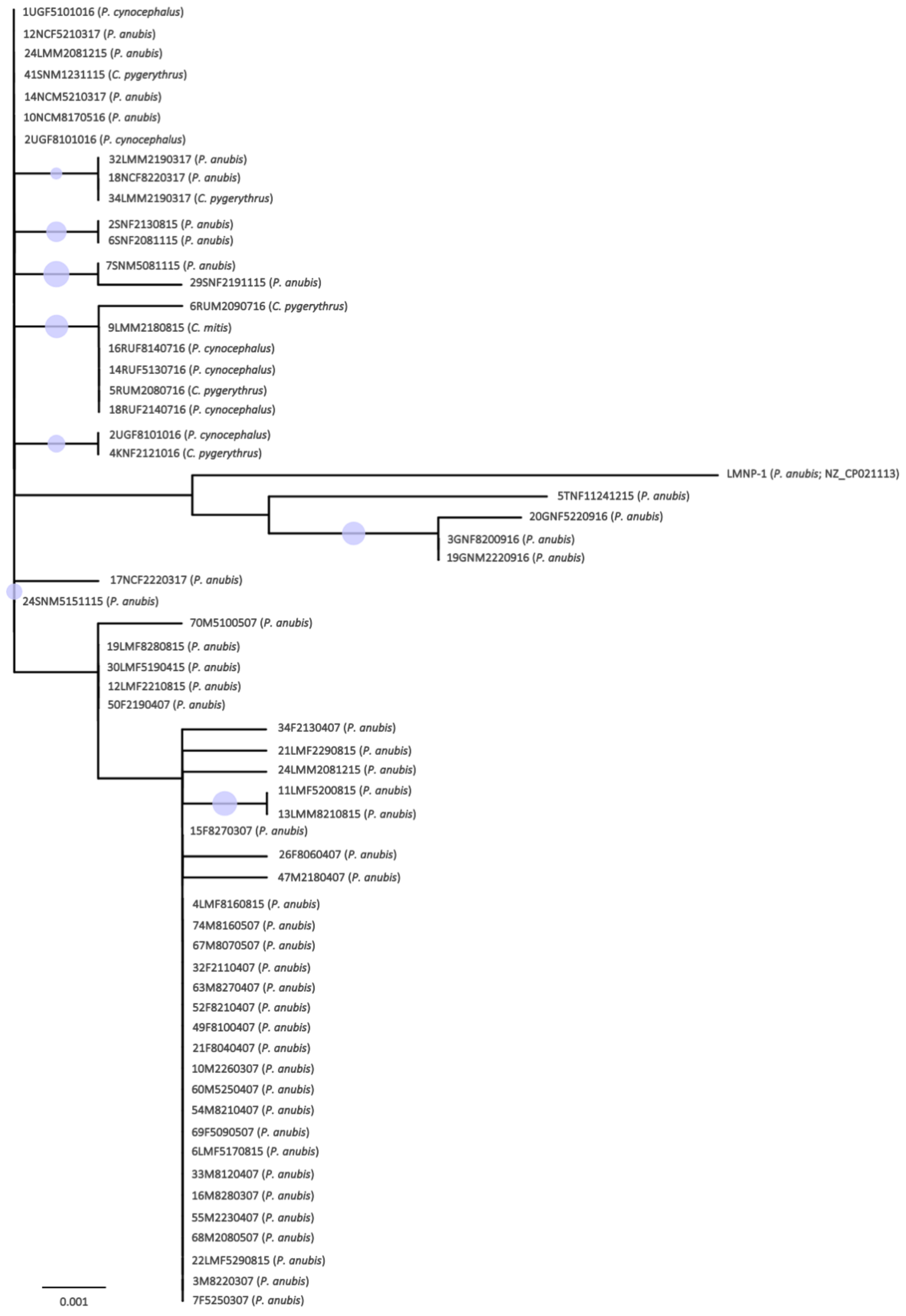

Figure S4. ML tree for the *TP0488* locus of the Tanzanian strains of NHP origin. 1,000 bootstrap replicates were performed and bootstrap values from 80-100% are highlighted as light blue circles of respective size. The bar indicates substitutions per site.

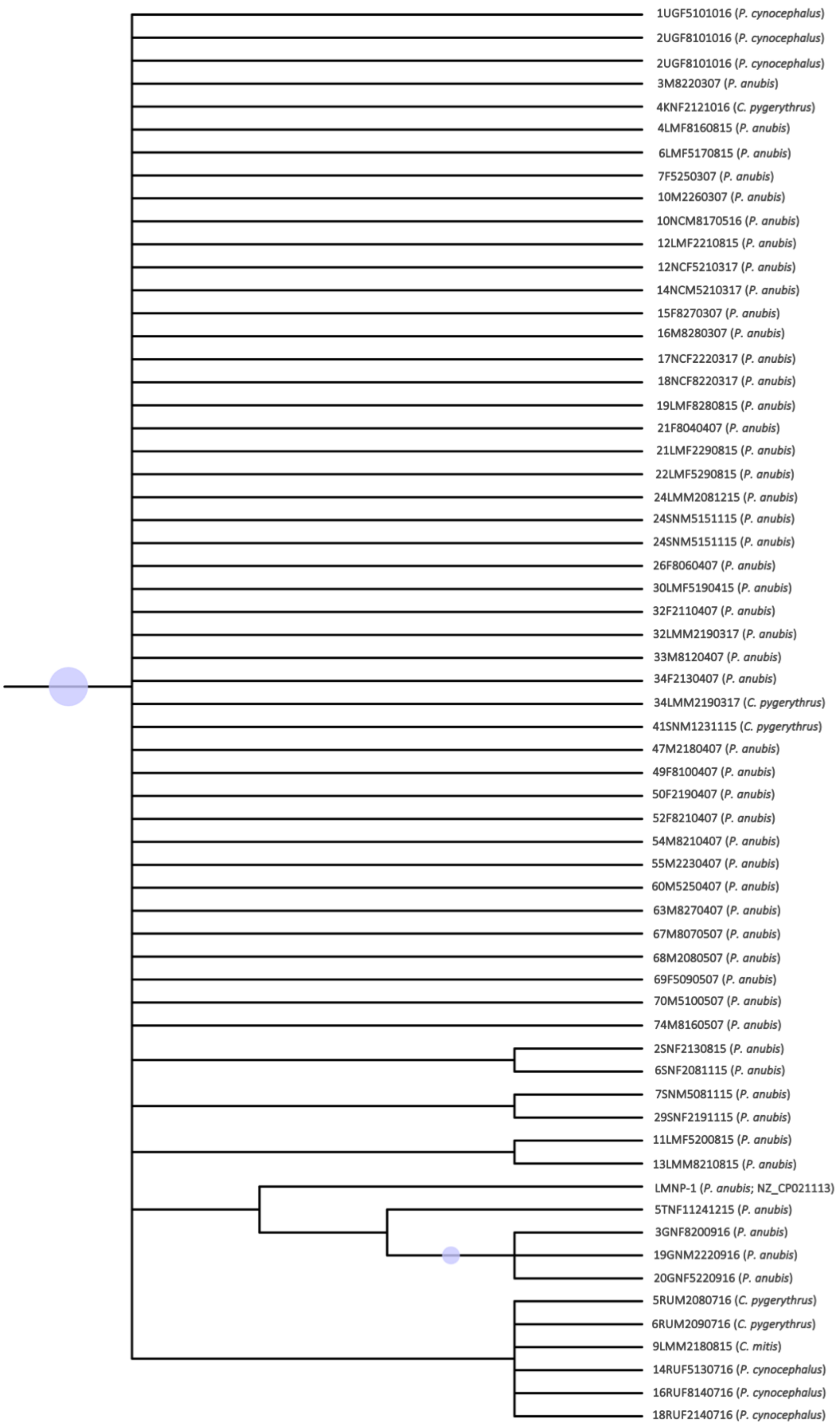

Figure S5. MP tree for the *TP0488* locus of the Tanzanian strains of NHP origin. 1,000 bootstrap replicates were performed and bootstrap values from 80-100% are highlighted as light blue circles of respective size. Gaps were coded as fifth character.

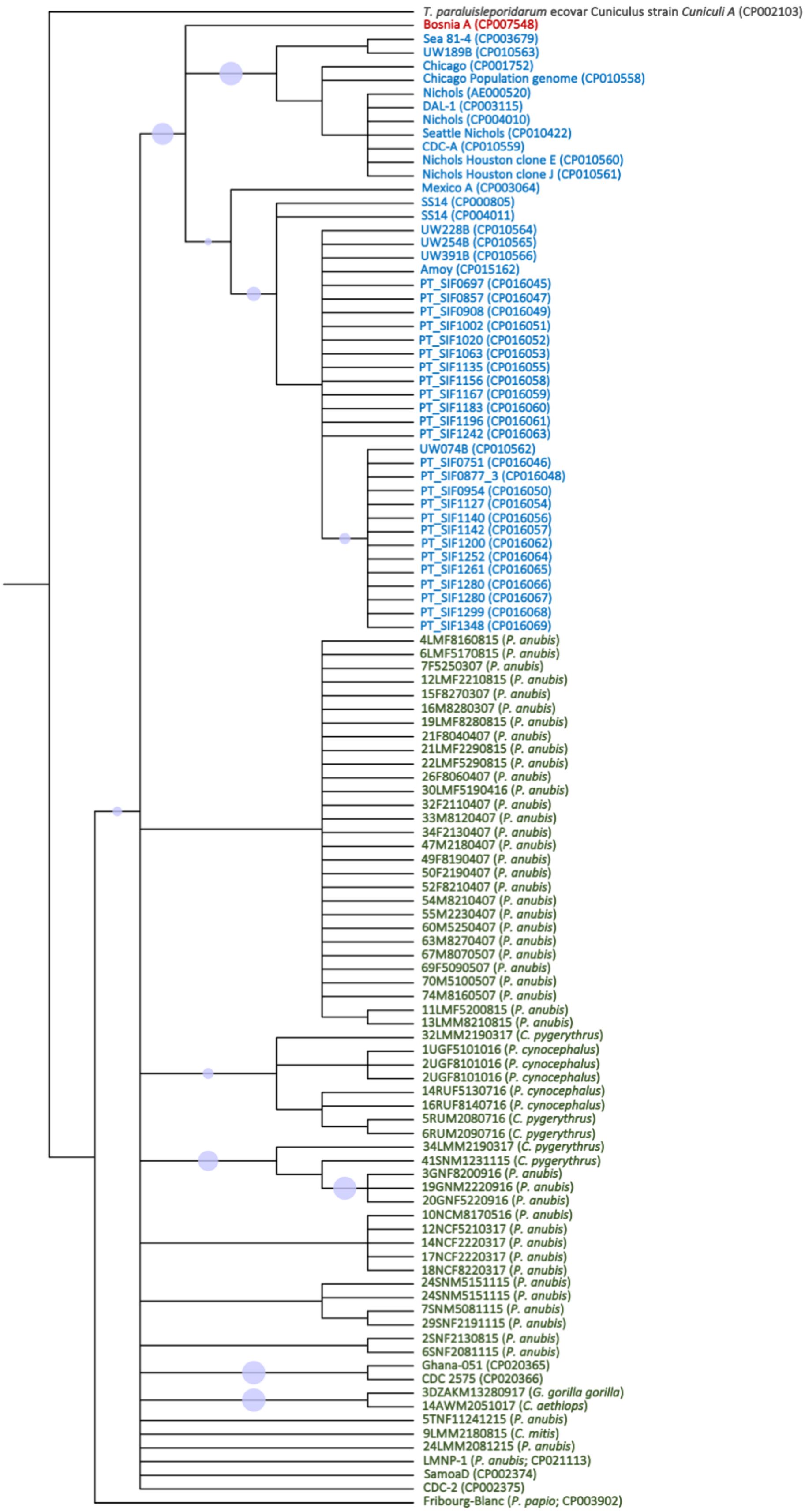

Figure S6. Rooted MP tree based on the concatenated sequences used for MLST (*TP0548* and *TP0488*). The tree is based on 1,773 nts and 1,000 bootstrap replicates.

Bootstrap values from 80-100% are highlighted as light blue circles of respective size. NHP species and/or GenBank accession numbers of published strains are provided in parentheses following the name of the strain. In all cases were the species is not mentioned, sequences are from *TP* of human origin. Blue=subsp. *pallidum*, green=subsp. *pertenue*, red=subsp. *endemicum*. The pathogen causing rabbit syphilis, *Treponema paraluisleporidarum* ecovar *Cuniculus* strain Cuniculi A, is used as an outgroup. The bar refers to substitutions per site. Gaps were coded as fifth character.

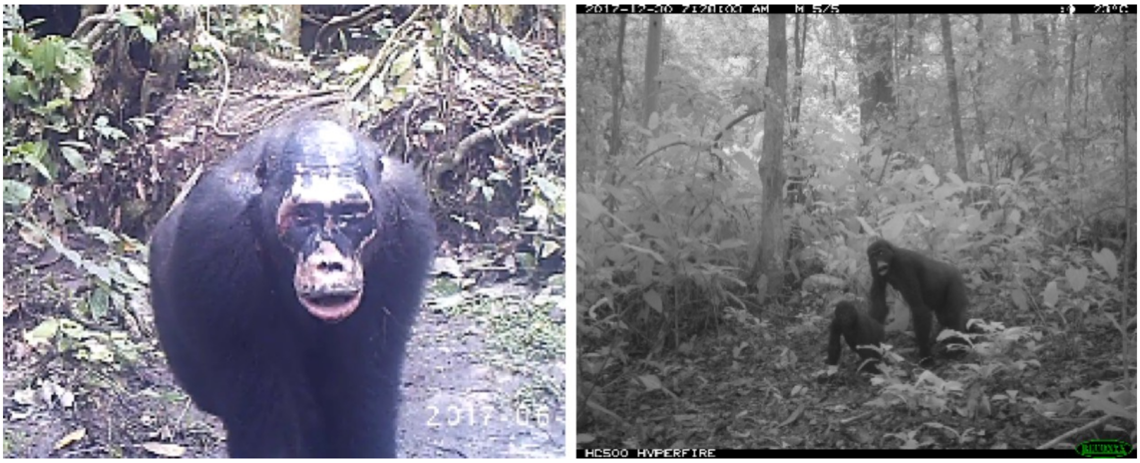

Figure S7. Camera trap photos of Western lowland gorillas with severe ulcerative skin lesions at Odzala-Kokoua National Park, Republic of the Congo, in 2017. Fecal samples analyzed in this study were collected in the same area and originated from animals with severe facial lesions. The images are not covered by the CC BY license. Image credits to African Parks (www.african-parks.org). All rights reserved, used with permission. When using the images online and across all platforms, African Parks and the photographer must be named appropriately, and African Parks and Odzala--Kokoua National Park should be mentioned using the following social media handles: on Twitter: @AfricanParks, Instagram: @AfricanParksNetwork and Facebook: @AfricanParks.Odzala--Kokoua National Park.

Supplementary References

37. Grillová, L., Noda, A. A., Lienhard, R., Blanco, O., Rodríguez, I. & Šmajs, D. Multilocus sequence typing of *Treponema pallidum* subsp. *pallidum* in Cuba from 2012 to 2017. *J Infect Dis*, jiy604 (2018).

38. Arora, N., Schuenemann, V., Jager, G., Peltzer, A., Seitz, A., Herbig, A., et al. Origin of modern syphilis and emergence of a pandemic *Treponema pallidum* cluster. *Nat Microbiol* **2**, 16245 (2016).

39. Flasarová, M., Pospíšilová, P., Mikalová, L., Vališová, Z., Dastychová, E., Strnadel, R., et al. Sequencing-based molecular typing of treponema pallidum strains in the Czech Republic: all identified genotypes are related to the sequence of the SS14 strain. *Acta Derm Venereol* **92**, 669-674 (2012).

40. Čejkova, D., Zobaníková, M., Chen, L., Pospíšilová, P., Strouhal, M., Qin, X., et al. Whole genome sequences of three *Treponema pallidum* ssp. *pertenue* strains: yaws and syphilis treponemes differ in less than 0.2% of the genome sequence. *PLoS Negl Trop Dis* **6**, e1471 (2012).

41. Zobaníková, M., Strouhal, M., Mikalová, L., Čejkova, D., Ambrožová, L., Pospíšilová, P., et al. Whole genome sequence of the *Treponema* Fribourg-Blanc: unspecified simian isolate is highly similar to the yaws subspecies. *PLoS Negl Trop Dis* **7**, e2172 (2013).

42. Strouhal, M., Mikalová, L., Haviernik, J., Knauf, S., Bruisten, S., Noordhoek, G. T., et al. Complete genome sequences of two strains of *Treponema pallidum* subsp. *pertenue* from Indonesia: Modular structure of several treponemal genes. *PLoS Negl Trop Dis* **12**, e0006867 (2018).

43. Marks, M., Fookes, M., Wagner, J., Butcher, R., Ghinai, R., Sokana, O., et al. Diagnostics for yaws eradication: Insights from direct next-generation sequencing of cutaneous strains of *Treponema pallidum*. *Clin Infect Dis* **66**, 818-824 (2018).
